# Individual copy number variation and extensive diversity between major MHC-*DAB1* allelic lineages in the European bitterling

**DOI:** 10.1007/s00251-021-01251-4

**Published:** 2022-01-11

**Authors:** Lorenzo Talarico, Anna Bryjová, Dagmar Čížková, Karel Douda, Martin Reichard

**Affiliations:** 1 Laboratory of Experimental Ecology and Aquaculture, Department of Biology, University of Rome Tor Vergata, Via Cracovia 1, 00133 Rome, Italy; 2 Italian National Institute for Environmental Protection and Research (ISPRA), Via Vitalino Brancati 60, 00144 Rome, Italy; 3 Institute of Vertebrate Biology, The Czech Academy of Sciences, Květná 8, 603 65 Brno, Czech Republic; 4 Department of Zoology and Fisheries, Czech University of Life Sciences Prague, Kamýcká 129, Prague, Czech Republic; 5 Department of Ecology and Vertebrate Zoology, Faculty of Biology and Environmental Protection, University of Łódź, Łódź, Poland; 6 Department of Botany and Zoology, Faculty of Science, Masaryk University, Brno, Czech Republic

**Keywords:** *Rhodeus amarus*, MHC class IIB, Positive selection, Gene duplication, MHC supertypes, Positively selected sites

## Abstract

Polymorphism of the major histocompatibility complex (MHC), *DAB1* gene was characterized for the first time in the European bitterling (*Rhodeus amarus*), a freshwater fish employed in studies of host-parasite coevolution and mate choice, taking advantage of newly designed primers coupled with high-throughput amplicon sequencing. Across 221 genotyped individuals, we detected 1–4 variants per fish, with 28% individuals possessing 3–4 variants. We identified 36 *DAB1* variants, and they showed high sequence diversity mostly located within predicted antigen-binding sites, and both global and codon-specific excess of non-synonymous mutations. Despite deep divergence between two major allelic lineages, functional diversity was surprisingly low (3 supertypes). Overall, these findings suggest the role of positive and balancing selection in promotion and long-time maintenance of *DAB1* polymorphism. Further investigations will clarify the role of pathogen-mediated selection to drive the evolution of *DAB1* variation.

**Supplementary information:**

The online version contains supplementary material available at 10.1007/s00251-021-01251-4.

## Brief communication

Bitterling fishes (Acheilognathinae) are a unique lineage of Cyprinid fishes distributed across the Palearctic region. Over the last decades, bitterling became widely used in evolutionary ecology research. Bitterling females lay their eggs into the gills of live freshwater mussels, and early offspring development is completed within the gill chamber (Reichard et al. 2007). In turn, host mussels parasitize fish (bitterling and other species) with free-swimming larvae (glochidia), which must attach to fish tissue and parasitize the fish to complete their development and start independent life as a juvenile mussel. Mussels have evolved various strategies to mitigate bitterling parasitism (Smith et al. 2004), and bitterling can reject parasitic mussel larvae (Douda et al. 2017). Ensuing a co-evolutionary arms race likely entails parasite-mediated selection. In addition, the bitterling mating system involves a strong role of female choice, particularly for the male phenotypic quality (body size, carotenoid-based colouration (Smith et al. 2004)) and genotype compatibility between partners at putatively adaptive genes of the major histocompatibility complex (MHC, see below), with major effects on offspring survival and fitness (Agbali et al. 2010; Reichard et al. 2012a).

MHC genes are ideal candidates to investigate parasite-mediated selection, given their involvement in the immune response of jawed vertebrates. Classical MHC class II genes encode for membrane-bound proteins recognizing antigens derived from extracellular parasites and pathogens, and presenting them to the immune system, hence triggering the immune cascade (Kelley et al. 2005). Because each MHC protein identifies a limited antigen array, intense positive selection promotes MHC sequence polymorphism, particularly at amino acid positions determining binding specificity (Radwan et al. 2020), the so-called antigen-binding sites (ABSs) that are mostly located in the exon2 of MHC class IIB genes (encoding the β1 domain). Sequence polymorphism eventually results in extreme allelic diversity per locus, further increased by frequent gene duplication and recombination events (Kaufman 2018), which should confer resistance against a broader range of antigens. Due to the adaptive nature of MHC, multiple mechanisms of pathogen-driven balancing selection and sexual selection are thought to be responsible for the persistence of extraordinary MHC variation over evolutionary time (Radwan et al. 2020).

In most Teleosts, the early whole-genome duplication has caused a complex evolutionary history of MHC genes, with events of gene duplication, rearrangement and loss generating extraordinary variability in architecture and diversity (Wang et al. 2017). Among classical genes of MHC class IIB, multiple *DAB*-like genes have been found across Cyprinids, as often deduced by sequence dissimilarity of detected variants (Seifertová and Šimková 2011 and references therein). Specifically, *DAB1* and *DAB3* genes were found in a few bitterling species (even though they often do not co-occur within individuals) to date, with the former usually showing a higher variability (Reichard et al. 2012a; Jeon et al. 2019; Won et al. 2021).

Here, we focused on MHC-*DAB1* gene in the European bitterling (*Rhodeus amarus*), a widely distributed species across most of Europe, where it comes into close contact with up to 16 species of unionid mussels and shows resistance to their glochidia to varying degrees at species and population levels (Reichard et al. 2012b; Douda et al. 2017). By amplifying the hypervariable exon2 with a newly designed primer pair coupled with next-generation amplicon sequencing, we aimed at characterizing *DAB1* architecture and polymorphism for the first time. Analyses of recombination, positive selection and phylogeny were also conducted to infer the evolutionary history of *DAB* genes in *R. amarus* and closely related Cyprinids.

We collected 221 *R. amarus* from 10 natural populations in the Czech Republic (Table 1) in August 2020 (authorizations MUBR 24,339/2018, MRS 31/2020 and CRS 1667/20). Fin clips were stored in absolute ethanol at – 20 °C until DNA extraction, which was carried out by Invisorb Spin Tissue Mini Kit following the producer protocol. Illumina compatible MHC amplicon libraries were prepared using two-step PCR. Each PCR product was barcoded by a combination of forward and reverse Nextera indexes and an inline barcode. For each individual, technical duplicates were prepared to account for noise due to PCR and sequencing stochasticity. The first PCR primers consisted of the newly designed forward (RamF-deg 5′-TTTCAGYTCATGGATASTAC-3′) and reverse (Rocel-rev 5′-CATGTGTGACAGGAGGATCAG-3′) MHC-*DAB1* primer sequences, flanked by 6 bp inline barcodes (in forward primers) and 33–34 bp long parts of Nextera adapters. First PCR was performed in 10 μl and included 1 × KAPA HIFI Hot Start Ready Mix (Kapa Biosystems, USA), each primer at 0.2 μM and 1 μl of DNA template under following conditions: initial denaturation at 95 °C for 3 min, followed by 28 cycles each of 95 °C (30 s), 55 °C (30 s) and 72 °C (30 s) and a final extension at 72 °C (5 min). Second PCR was used to reconstruct dual indexed Nextera sequencing adaptors and was performed in 15 µl using 1 × KAPA HIFI Hot Start Ready Mix (Kapa Biosystems, USA), each Nextera primer at 2 μM and 1 μl of the first PCR product as template. Conditions of the second PCR were as the first one, except that the number of PCR cycles was twelve. PCR products of the second PCR were quantified by 1.5% agarose gel and pooled equimolarly. The final library was cleaned up using Agencourt AmpureXP beads (Beckman Coulter Life Sciences). Products of the desired size (380–520 bp) were extracted by PipinPrep (Sage Science Inc., USA) and sequenced on Illumina Miseq (v3 kit, 300 bp paired-end reads) at the Central European Institute of Technology (CEITEC), Masaryk University, Brno (Czech Republic).

**Table 1:** Geographic information for 10 sampling locations (Pop) of *R. amarus* in the Czech Republic, along with the number of collected fish (*N*), geographic location (latitude, longitude), the number of MHC-*DAB1* variants (A) and *DAB1* allelic richness (Ar, the average number of variants present in 1000 random samples of 19 fishes drawn from the same population, with one Standard Deviation). The datum for geographic coordinates is WGS84

Pop	N	Location	River catchment	Drainage basin (drainage sea)	Lat° (N)	Lon° (E)	A	Ar (± SD)
S01	22	Zidlochovice	Dyje	Danube (Black Sea)	49.040	16.616	12	11.7 (± 0.5)
S03	26	Lednice	Dyje	Danube (Black Sea)	48.818	16.809	18	15.8 (± 1.4)
S04	23	Hradisko	Morava	Danube (Black Sea)	49.329	17.357	18	16.7 (± 1.1)
S05	21	Otrokovice	Morava	Danube (Black Sea)	49.204	17.512	10	9.7 (± 0.5)
S06	23	Mikovice	Morava	Danube (Black Sea)	49.041	17.504	13	12.3 (± 0.9)
S07	19	Bohumin	Oder	Oder (Baltic Sea)	49.915	18.321	17	17 (± 0)
S08	19	Dehylov	Oder	Oder (Baltic Sea)	49.884	18.173	4	4 (± 0)
S09	19	Studenka	Oder	Oder (Baltic Sea)	49.701	18.066	7	7 (± 0)
S10	24	Valy	Elbe	Elbe (North Sea)	50.034	15.616	8	7.5 (± 0.9)
S11	25	Dasice	Elbe	Elbe (North Sea)	50.046	15.887	6	5.8 (± 0.4)

Bioinformatic data pre-processing involved de-multiplexing and removal of primer and inline barcode sequences at 5′ ends of the reads with Skewer (Jiang et al. 2014), removal of 3′ primers and read-through adapters with NGmerge (Gaspar 2018), quality filtering (i.e. truncating reads at 200 bp, removing reads with low quality or containing ambiguous bases) with DADA2 (Callahan et al. 2016) and paired reads merging with FLASH (Magoč and Salzberg 2011). Successively, we used AmpliCHECK (Sebastian et al. 2016) to visually inspect our data and explore the frequency of putative artefacts. We observed that variants showing a per-amplicon frequency (PAF) < 5.5% were identified as chimaeras or low-frequency sequencing/PCR errors (namely variants showing 1–2 substitutions from putative true parental variants with higher PAF within the same amplicon), while variants likely originated by tag-switching/cross-contamination from other samples occurred with < 3% PAF. Therefore, to obtain the final set of genotypes, we (1) filtered out variants showing PAF < 5.5% and (2) retained only alleles found in both PCR replicates.

MHC variants were aligned in MEGAX (Kumar et al. 2018) using the MUSCLE algorithm. Possible recombination breakpoints in the alignment were explored with the GARD method (Kosakovsky Pond et al. 2006) implemented in the Datamonkey server (http://www.datamonkey.org/). To depict genealogical relationships among *R. amarus DAB1* variants, we built a neighbour-net network (Kimura 2-Parameter distances) in SplitsTree5 (Huson and Bryant 2006), since it allows to visualize conflicting signals due to gene duplication and/or putative recombination events. To offer a more complete evolutionary picture, we also built a second network including additional 90 exon2 variants of MHC-*DAB1* and *DAB3* genes from related Cyprinids: 17 of *Rhodeus ocellatus* (276 bp), 26 of *Rhodeus pseudosericeus* (210 bp), 21 of *Rhodeus sinensis* (273 bp) representative of major allelic lineages, 16 of *Pseudorhodeus tanago* (274 bp) and 10 of *Squalius cephalus* (276 bp) — aligned sequences and GenBank accessions are provided in Supplementary data S1.

**Fig. 1:**
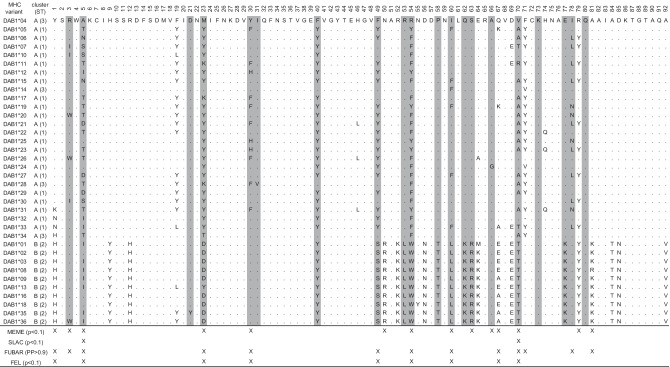
Amino acid alignment of 36 MHC-*DAB1* variants in 221 *R. amarus* from the Czech Republic. The allelic lineage (cluster) and supertype (ST) are provided for each MHC-*DAB1* variant. Grey-shaded positions identify predicted ABS according to human MHC. “X” signs mark codons under positive selection (PSS) as inferred by each of four performed tests (*p: p*-value; PP: posterior probability)

Measures of sequence polymorphism, namely the average pairwise nucleotide (Kimura 2-Parameter model) and amino acid (Poisson correction model) distances, were computed in MEGAX in three alignment partitions: (i) all sites, (ii) 20 predicted ABS based on human MHC (Brown et al. 1993) (Fig. 1), (iii) non-ABS. We verified the signature of historical positive selection testing (one-tailed Z-test in MEGAX) for an excess of non-synonymous (dN) substitutions over synonymous substitutions (dS) through the modified Nei-Gojobori method (with Jukes-Cantor correction and 1000 bootstrap replicates) in the three above-mentioned partitions separately. We also identified positively selected sites (PSS) according to four methods implemented in Datamonkey — the single-likelihood ancestor counting (SLAC), the fixed effects likelihood (FEL), the fast-unconstrained Bayesian approximation (FUBAR), and the mixed-effects model of evolution (MEME) (Kosakovsky Pond and Frost 2005; Murrell et al. 2012, 2013).

Finally, variants were grouped into (theoretical) immunological supertypes based on similarity of physicochemical properties at predicted ABS (Schwensow et al. 2007), following the statistical procedure of Doytchinova and Flower (2005). In brief, ABSs were extracted, and each site was characterized according to five z-descriptors as in Sandberg et al. (1998). Then, the K-means clustering algorithm implemented in the adegenet R-package (Jombart 2008) was used to group variants into supertypes. The ΔBIC > 2 criterion (i.e. the last increase in K to reduce BIC by > 2) was adopted to choose the optimal number of supertypes.

After data pre-processing, the average amplicon coverage was 14,577 reads (± 5867 SE). All genotypes were consistent between replicates after the frequency-based filtering step, i.e., the same variants were found in both PCR duplicates, so that the second genotyping step actually did not contribute to adjust the final genotype set. Individuals showed 1–4 variants each that cumulatively represented on average 70.2% (± 4.8% SE) of the amplicon coverage. We gathered 36 MHC-*DAB1* variants (GenBank accessions: OL840346-OL840381) of 277 bp or 274 bp (i.e. DAB1*32) translating into unique amino acid sequences (Fig. 1) with no stop codons in the reading frame. No recombination breakpoints in the global alignment were detected by GARD.

**Fig. 2:**
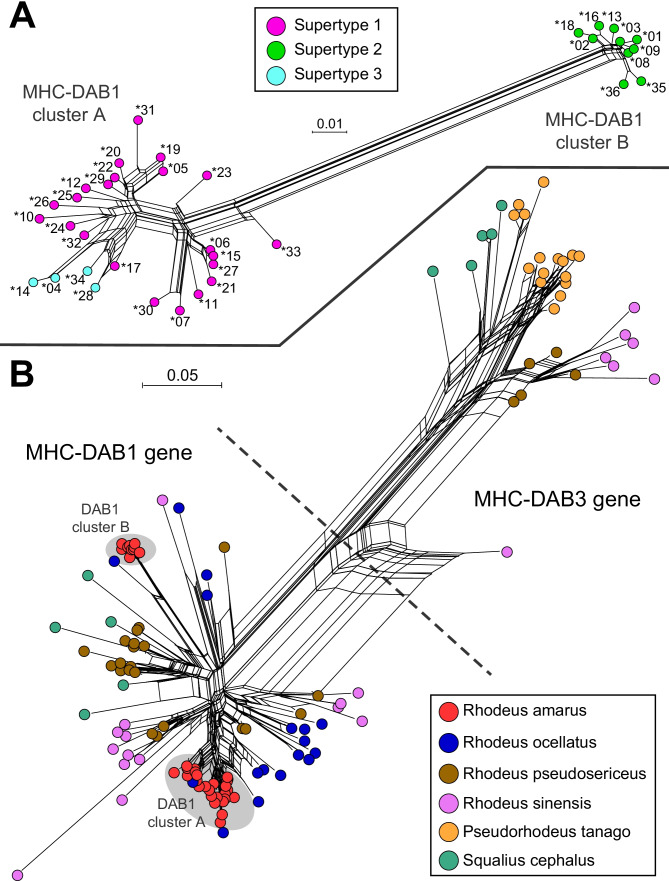
Neighbour-net networks (based on Kimura 2-Parameter distances) depicting the relationship among: **A** 36 *R. amarus* MHC-*DAB1* variants; **B** overall 126 MHC-*DAB1* and *DAB3* variants from six Cyprinids (supplementary data S1). Dot colours denote MHC-*DAB1* supertypes in **A**, and species in **B**. The two major *R. amarus DAB1* allelic lineages, namely cluster A and B, are indicated in both networks. Codes of MHC-*DAB1* variants in (A) refer to those in Fig. 1

**Fig. 3:**
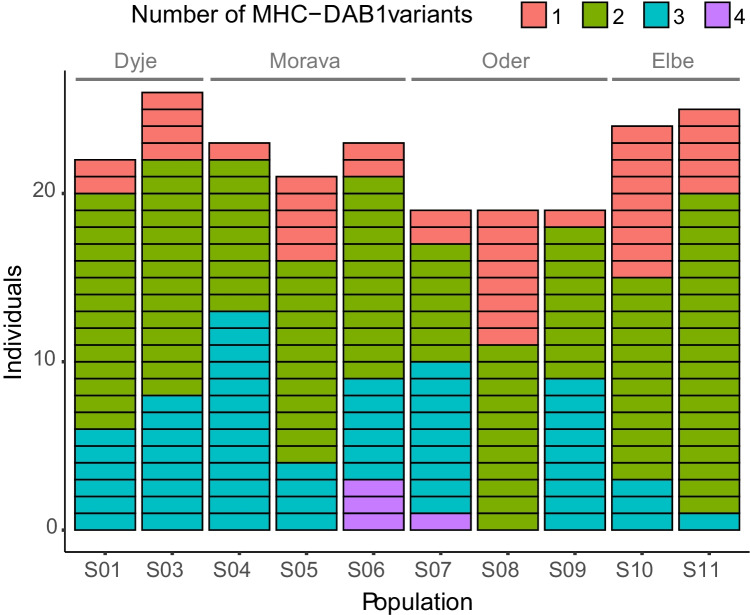
The number of individuals carrying 1, 2, 3 or 4 MHC-*DAB1* variants across 10 examined populations of *R. amarus* from four river catchments in the Czech Republic. Population codes refer to those in Table 1

In the Neighbour-net network, *R. amarus DAB1* variants formed two clear phylogenetic groups of 26 and 10 variants (PERMANOVA based on pairwise nucleotide distances: *F* = 237.4; *p* = 0.0001; 9999 permutations). We refer to them as cluster A and cluster B (Fig. 2A). These may correspond to two *DAB1*-like genes, at least partly independently evolved and with no shared alleles. Note that they do not correspond to the *DAB1* and *DAB3* MHC genes of Cyprinids, as evident from the phylogenetic network in Fig. 2B. The occurrence of no more than two alleles per individual per putative gene could support such a hypothesis. On the other hand, 55 and 38 individuals scattered across populations showed no amplification/missing gene for the first and the second hypothetical *DAB* gene. Furthermore, when testing for deviations from Hardy–Weinberg expectations, overall 4 and 5 populations revealed a strong heterozygote deficiency (*p* < 0.05 after Bonferroni correction) for the former and the latter putative gene, respectively (results not shown). For such reasons, the hypothesis of two independently evolved and substantially diverged MHC-*DAB1*-like genes (i.e. substantially diverged paralogues) does not appear convincing. In consequence, we conservatively assumed that phylogenetic groups correspond to deeply diverged allelic lineages within the *DAB1* gene, rather than two separate loci. Anyhow, the occurrence of individuals carrying > 2 *DAB1* variants (28.5%) implies the frequent duplication of the *DAB1* gene. Furthermore, if the duplication event is relatively recent, which appears likely, the same alleles can occur in both gene copies, and we cannot assign alleles to loci unequivocally. This likely underestimates the occurrence of duplication across individuals. Alternative methods, such as Southern Blotting and long-read sequencing, should be considered for future in-deep investigations on the genetic architecture of class II MHC genes. The distribution of the number of variants carried by an individual across populations is shown in Fig. 3.

Considering all variants, both nucleotide and amino acid diversity were at least fourfold higher in ABS than non-ABS sites, and non-synonymous substitutions exceeded the synonymous ones with statistical significance in all examined partitions (Table 2). Cluster B revealed lower sequence polymorphism and less pronounced dN > dS compared to cluster A. Overall, 17 putative PSS were identified, 11 of which were supported by ≥ 2 methods (Fig. 1). The supertyping procedure allowed grouping variants into three functional supertypes: the Supertype-2 (10 variants) perfectly matched with cluster B, while Supertype-1 and -3, respectively, included 22 and 4 variants from cluster A (Figs. 1 and 2A).

**Table 2:** Sequence polymorphism of *R. amarus* MHC-*DAB1* variants (within two major allelic lineages separately and overall) for each of three alignment partitions: 20 sites corresponding to human ABSs, non-ABSs, all sites. The average nucleotide (Kimura 2-Parameter) and amino acid (Poisson corrected) distance with the standard error (SE), and the *Z*-statistic for the test of dN > dS with the corresponding *p*-value (significant ones are in bold)

Group	No. of sequences	Partition	Nucleotide distance (± SE)	Amino acid distance (± SE)	*Z*	*p*-value
***DAB1*-cluster A**	26	ABS	0.134 (0.029)	0.298 (0.084)	2.508	**0.007**
		Non-ABS	0.021 (0.006)	0.038 (0.014)	1.489	0.070
		all	0.044 (0.008)	0.088 (0.020)	2.912	**0.002**
***DAB1*-cluster B**	10	ABS	0.026 (0.014)	0.055 (0.029)	1.496	0.069
		Non-ABS	0.010 (0.004)	0.023 (0.012)	0.993	0.161
		all	0.013 (0.004)	0.030 (0.011)	1.875	**0.032**
**Overall**	36	ABS	0.243 (0.046)	0.552 (0.132)	3.027	**0.002**
		Non-ABS	0.058 (0.011)	0.119 (0.028)	2.128	**0.018**
		all	0.093 (0.013)	0.194 (0.033)	3.500	**0.000**

Our most apparent finding is the duplication of the *DAB1* gene in a substantial proportion of individuals, indicating frequent individual copy number variation, with no apparent geographical pattern (Fig. 3). Concerning *DAB1* of related Cyprinids, multiple candidate loci were inferred strictly through sequence similarity in *R. sinensis* (Jeon et al. 2019), while a single locus was apparently amplified in *R. ocellatus* (Agbali et al. 2010; Reichard et al. 2012a) and *S. cephalus* (Seifertová and Šimková 2011). However, different MHC amplification and genotyping strategies were employed in the previous and current study, possibly leading to a lower comparative power between results.

The *DAB1* in *R. amarus* showed remarkable allelic (36 variants) and sequence (40.2% of polymorphic amino acid sites) diversity, a surplus of non-synonymous mutations especially at sites putatively involved in the peptide binding, and PSS mostly matching (64.7%) with predicted ABS corresponding to human MHC (*p*-value < 0.0001, Fisher’s exact test). Altogether, these outcomes support either the functionality of the gene or the central role of (historical) positive selection to promote *DAB1* diversity through amino acid replacement, as expected for classical MHC genes (Radwan et al. 2020). Interestingly, supposed functional diversity, unlike sequence and allelic diversity, was surprisingly reduced: only three supertypes were inferred, and all variants of the highly diverged cluster B were predicted to be functionally similar (all belong to supertype-2). Moreover, we found dN > dS at ABS without a rigorous statistical support (i.e., *p* = 0.07: Table 2) within such allelic lineage. This pattern may indicate relaxed positive selection in the B cluster, though it could be also imposed by difference in the identity of PSS between A and B, with cluster A positively selected sites better matching the predicted ABS. Also, we cannot entirely rule out the possibility that low number of analysed cluster B sequences (10) and their low diversity may have affected the test power. To further explore the nature of this pattern, we ran the aBSREL analyses (Smith et al. 2015) to test for episodic dN > dS over branches, specifically selecting the branch connecting cluster A to cluster B variants. We found strong statistical support for the “full adaptive” model considering diversifying selection acting along this branch (dN/dS = 4.43; *p* < 0.00001), compared to the baseline neutral model. The full adaptive model showed two classes of sites with different evolutionary modes: 78% of sites under neutral evolution (dN/dS = 1) and 22% of sites under strong diversifying selection (dN/dS = 73.7). This indicates that the divergence between the two clusters at approximately 1/5 of the sites was due to strong positive selection and supports the high functional divergence between the variants from different clusters. We acknowledge that experimental support is needed to establish the (adaptive) significance of such a pattern of extreme inter-lineage sequence diversity coupled with low intra-lineage functional diversity. Further, comparisons of the *DAB1* polymorphism degree among species cannot be done accurately because of different gene architecture (1–6 putative loci) as well as disparate sampling designs and/or genotyping approaches adopted in previously published studies. Yet, the overall *R. amarus DAB1* variability appeared lower compared to that found in related Cyprinids, such as *S. cephalus* (45 alleles in 191 fishes from 14 populations representative of the whole species range; nucleotide diversity = 0.138; amino acid diversity = 0.262; Seifertová and Šimková 2011), *R. sinensis* (104 alleles in 50 fishes form 10 populations; Jeon et al. 2019) or *R. ocellatus* (overall 33 alleles in 112 captive-bred individuals cumulating samples from Agbali et al. (2010) and Reichard et al. (2012a)). Finally, even if only 21 alleles were found in 222 *R. pseudosericeus* individuals from 7 wild populations, they could be grouped into 7 functional supertypes (Won et al. 2021).

Phylogenetic analyses (Fig. 2) revealed further features of the *DAB* gene evolution that are typical of MHC genes. First, complex (unresolved) genealogies among variants were depicted, indicating the intricate evolution by reticulation. We did not detect evidence of recombination in examined *R. amarus* sequences, which may indicate its negligible contribution to generating *DAB1* diversity — note that the same was found in *S. cephalus* (Seifertová and Šimková 2011), but see Jeon et al. (2019) for the contrary in *R. sinensis*. Second, sequence similarity among variants of different species was more pronounced than that between variants of a single species in some cases, resulting in phylogenetic clusters including variants from multiple species (e.g. *R. amarus* and *R. ocellatus*, or *R. sinensis* and *R. pseudosericeus*). This phenomenon, referred to as trans-species polymorphism (Klein 1987), indicates that MHC polymorphism is ancient in this taxon and has been maintained for evolutionary times even predating speciation events (Klein et al. 2007) — for instance, the estimated divergence between *R. amarus* and *R. ocellatus* dates about 15–16 Mya (Cheng et al. 2014). Accordingly, previous studies targeting *DAB* genes revealed trans-species polymorphism occurring within and between subfamilies of Cyprinids (Ottová et al. 2005; Jeon et al. 2019).

The long-time maintenance of multiple deeply diverged allelic lineages and, by extension, the occurrence of the trans-species polymorphism likely result from balancing selection, although these could be explained even by the interplay of other mechanisms, such as adaptive introgression or mate choice for dissimilar MHC alleles (Radwan et al. 2020). The contribution of the latter was indeed detected for the congeneric *R. ocellatus* (Agbali et al. 2010; Reichard et al. 2012a); nevertheless, the involvement of pathogen-mediated balancing selection processes (e.g. heterozygote advantage, divergent allele advantage, frequency-dependent selection) has not been specifically addressed to date in bitterling fishes.

In conclusion, our study provides the first characterization of MHC-*DAB1* in the European bitterling, a model used in studies of host-parasite coevolution and mate choice, where MHC diversity is under selection. We estimated genetic diversity and clarified, at least in part, the gene structure and evolution. Mechanisms involved in maintaining the observed deep divergence between two major allelic lineages, as well as the adaptive significance of the substantial polymorphism that apparently does not correspond to elevated levels of functional diversity, are important questions deserving further investigations.

## Supplementary Information

Below is the link to the electronic supplementary material.

Supplementary file1 (DOCX 24 KB)

Supplementary file2 (NEX 43 KB)
