# Supplementary material for: Individual copy number variation and extensive diversity between major MHC-DAB1 allelic lineages in the European bitterling
Source: Immunogenetics. 2022 Jan 11;74(5):497–505. doi: 10.1007/s00251-021-01251-4 (PMC9467946; doi:10.1007/s00251-021-01251-4)
Supplement: Supplementary file 1 — Supplementary file1 (DOCX 24 KB) [file 251_2021_1251_MOESM1_ESM.docx]

Supplementary data S1

Nucleotide alignment (Nexus format) of overall 126 MHC-*DAB1* and *DAB3* alleles from the present study (*R. amarus*, 36 sequences) and the literature (*Rhodeus ocellatus* , 17 sequences; *Rhodeus pseudosericeus,* 26 sequences*;* *Rhodeus sinensis* , 21 sequences; *Pseudorhodeus tanago* , 16 sequences; *Squalius cephalus* , 10 sequences) used for the phylogenetic reconstruction. The GenBank accession and the allele identifier are provided for each sequence.
